# Long non-coding RNA NEAT1 promotes non-small cell lung cancer progression through regulation of miR-377-3p-E2F3 pathway

**DOI:** 10.18632/oncotarget.10108

**Published:** 2016-06-16

**Authors:** Chengcao Sun, Shujun Li, Feng Zhang, Yongyong Xi, Liang Wang, Yongyi Bi, Dejia Li

**Affiliations:** ^1^ Department of Occupational and Environmental Health, School of Public Health, Wuhan University, Wuhan, P. R. China; ^2^ Wuhan Hospital for the Prevention and Treatment of Occupational Diseases, Wuhan, P. R. China

**Keywords:** long non-coding RNA NEAT1 (lncRNA NEAT1), hsa-miRNA-377-3p (miR-377-3p), E2F3, non-small cell lung cancer (NSCLC), tumorigenesis

## Abstract

Recently, the long non-coding RNA (lncRNA) NEAT1 has been identified as an oncogenic gene in multiple cancer types and elevated expression of NEAT1 was tightly linked to tumorigenesis and cancer progression. However, the molecular basis for this observation has not been characterized in progression of non-small cell lung cancer (NSCLC). In our studies, we identified NEAT1 was highly expressed in patients with NSCLC and was a novel regulator of NSCLC progression. Patients whose tumors had high NEAT1 expression had a shorter overall survival than patients whose tumors had low NEAT1 expression. Further, NEAT1 significantly accelerates NSCLC cell growth and metastasis in vitro and tumor growth in vivo. Additionally, by using bioinformatics study and RNA pull down combined with luciferase reporter assays, we demonstrated that NEAT1 functioned as a competing endogenous RNA (ceRNA) for hsa-miR-377-3p, antagonized its functions and led to the de-repression of its endogenous targets E2F3, which was a core oncogene in promoting NSCLC progression. Taken together, these observations imply that the NEAT1 modulated the expression of E2F3 gene by acting as a ceRNA, which may build up the missing link between the regulatory miRNA network and NSCLC progression.

## INTRODUCTION

Lung cancer is the main cause of cancer-related deaths both in men and women around the world for several decades [1, 2]. There are approximately 1.8 million new cases to be in morbidity in 2012 (12.9% of the total), killing about 1.59 million (19.4% of the total) people per year globally [1, 2]. There are about 80% of lung cancers classifying histopathologically as non-small cell lung carcinomas (NSCLC). At early stages of NSCLC, the only treatment is surgery, but the 5-year overall survival (OS) rate is only about 40% [3, 4], whereas chemotherapy is mostly employed for small cell lung cancer (SCLC). Dysregulation of proto-oncogenes and silencing of tumor suppressor genes contribute to the initial, development and progression of NSCLC [5–10].

Long non-coding RNAs (lncRNAs) are a class of non-coding RNAs, whose length is more than 200 nucleotides (nt) [11, 12]. They have been formed as a novel field of biology, with increasing evidence indicating that they are often with cell-type feature, and contribute critical roles on numerous of systems and might make sense with known cancer genes [12–14]. A handful of studies [15–28] have found that lncRNAs could be important players in cancer biology, especially resulting in aberrant expression of gene products that contribute to the advance of numerous of human tumors [29–31]. Additionally, lncRNAs expression could be also used as diagnostic or prognostic markers on account of it may confer clinical characteristics about tumor outcomes [22, 25, 28, 32]]. Nevertheless, the clinical significance and biological mechanisms of lncRNAs in the progression of NSCLC remain largely unknown.

Recent reports have suggested that crosstalk between architectural features of nuclear bodies and ncRNAs results in the accurate control of its target gene expression [33, 34]. NEAT1 (nuclear enriched abundant transcript 1) has two isoforms: NEAT1_1 (3.7 kb) and NEAT1_2 (23 kb) [33, 34]. It was recently demonstrated as a crucial architectural component of a paraspeckle structure [34–38]. NEAT1 plays roles on controlling numerous of biological processes, including cellular differentiation and stress response through paraspeckles pathway [34, 39–41]. Moreover, NEAT1 also positively correlates with poor survival in breast cancer patients [42]. NEAT1 has also been found to favor oncogenic gene transcription by altering epigenetic landscape of its promoters, resulting in driving cell growth in prostate cancer [43]. These results suggest tumor-oncogenic functions of NEAT1 on NSCLC but as of now this assumption has not been well investigated.

In our study, we aimed to discover another underlying molecular mechanism of lncRNA NEAT1 on NSCLC progression. Using starBase2.0 software (http://starbase.sysu.edu.cn/seedTargetInfo.php?type=lncRNA&database=hg19&name=hsa-miR-377-3p&geneName=NEAT1&autoId=3608&orgTable=mirLncRNAInteractionsAll), we identified NEAT1 (NEAT1_2, NR_131012, and we recognized NEAT1_2 as a representation of NEAT1 in our research) harbors three conserved miR-377-3p cognate sites, and we made a hypothesis NEAT1 might function as a competing endogenous RNA (ceRNA) for miR-377-3p. Then, we searched different miRNA-target gene predicted data bases (including microRNA.org, TargetScan, and PicTar) to seek potential targets of miR-377-3p that exhibited oncogenic properties, and found oncogene E2F3 harbors two conserved miR-377-3p cognate sites and is a predicted target of miR-377-3p. Corporately, it is concluded that NEAT1 could be a crucial oncogenic regulator involved in NSCLC tumorigenesis and progression through acting as a ceRNA for miR-377-3p, and in return activation of E2F3 pathway.

## RESULTS

### NEAT1 is up-regulated in NSCLC lung tissues and cell lines, and correlates with poor prognosis

**Figure 1:**
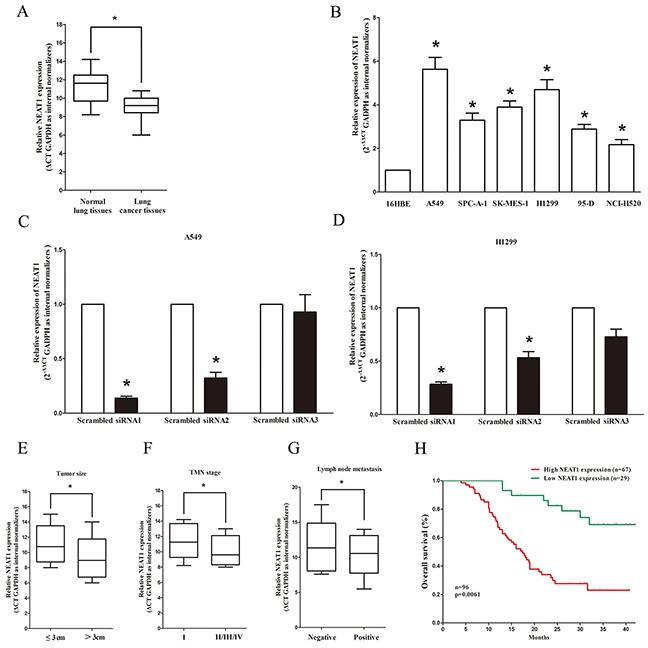
Relative NEAT1 expression in non-small cell lung cancer tissues and cell lines, and its clinical significance **A.** Relative expression of NEAT1 expression in NSCLC tissues (n = 96) and in paired adjacent normal tissues (n = 96). NEAT1 expression was examined by qPCR and normalized to GAPDH expression. (shown as ΔCT). **B.** Relative expression of NEAT1 expression in NSCLC cell lines and normal HELF lung epidermal cell. **C-D.** Relative NEAT1 expression in A549 and H1299 cells after transfecting with si-NEAT1, namely, siRNA1, siRNA2 and siRNA3. NEAT1 expression was examined by qPCR and normalized to GAPDH expression (shown as 2^−ΔΔCT^). **E-G.** NEAT1 expression was significantly higher in patients with big tumor size, advanced clinical stage and lymph nodes metastasis. NEAT1 expression was examined by qPCR and normalized to GAPDH expression. (shown as ΔCT). **H.** The Kaplan-Meier survival analysis indicated that NEAT1 high expression (red line, n=67) has a worse overall survival compared to the low expression subgroup (green line, n=29). *P < 0.05. Means ± SEM are shown. Statistical analysis was conducted using student t-test.

To verify if NEAT1 was differentially expressed in NSCLC tissues, 96 paired NSCLC lung tissues and their pair-matched adjacent normal lung tissues were tested for NEAT1 expression. AS expect, NEAT1 expression was remarkably increased in cancerous tissues than that of in normal counterparts (Figure 1A). Further, we also detected NEAT1 expression in cell lines, and results indicated NEAT1 was higher expressed in NSCLC cell lines, including A549, H1299, SK-MES-1, SPCA-1, 95-D, and NCI-H520 cell lines, than normal 16HBE (human bronchial epithelial) cell (Figure 1B). Small interfering RNAs (siRNAs) assay was used to down-regulate NEAT1 expression in NSCLC cells (A549 and H1299),. At forty-eight hours of post-transfection, NEAT1 expression was knocked down by approximately 80% in A549 and H1299 cells by si-NEAT1(siRNA1) transfection when compared with the scrambled siRNA (Figure 1C-1D). Moreover, to assess the clinical significance of NEAT1, we evaluated the association between its expression and clinic-pathological parameters (including lymph node metastasis, maximum diameter, and stage and so on). Results revealed NEAT1expression in NSCLC lung tissues were remarkably corrected with TNM stage (*P* = 0.0014), tumor size (*P* = 0.0006), and lymph node metastasis (*P* < 0.001). Nevertheless, NEAT1 expression was not associated with age (*P* = 0.2912), gender *(P* = 0.3893), differentiation (*P* = 0.3066), and histological tumor type (*P* = 0.1532) (Figure 1E-1G, Table 1). In addition, high NEAT1 expression levels in patients with NSCLC (>2 folds of increase, n=67) had a shorter overall survival than that of with low NEAT1 expression levels (≤2 folds of increase, n=29 (Figure 1H), indicating by Kaplan–Meier survival analysis. These results demonstrated that high expression levels of NEAT1 were associated with poor prognosis.

**Table 1:** Correlation between NEAT1 expression and clinicopathological parameters of NSCLC patients(n=96)

Parameter	n	Relative NEAT1 expression		
		Low	High	*P*-value[^a^]
Age/years				0.2912
≤ 65	62	21	41	
> 65	34	8	26	
Gender				0.3893
Male	60	20	40	
Female	36	9	27	
Differentiation				0.3066
Well, moderate	52	18	34	
Poor	44	11	33	
Tumor size (maximum diametercm)				0.0006[*]
≤ 3cm	41	20	21	
> 3cm	55	9	46	
Smoking history				0.6216
Smokers	73	23	50	
Never smokers	23	6	17	
Lymph node metastasis				0.001[*]
Positive	57	7	50	
Negative	39	22	17	
TMN stage				0.0014[*]
I	28	15	13	
II/III/IV	68	14	54	
Histological tumor type				0.1532
Squamous cell carcinoma	21	9	12	
Adenocarcinoma	75	20	55	

a Chi-square test

* P < 0.05

These data demonstrate that the up-regulation of NEAT1 may play important roles on NSCLC development and progression.

### NEAT1 promotes tumor NSCLC cell growth in vitro

**Figure 2:**
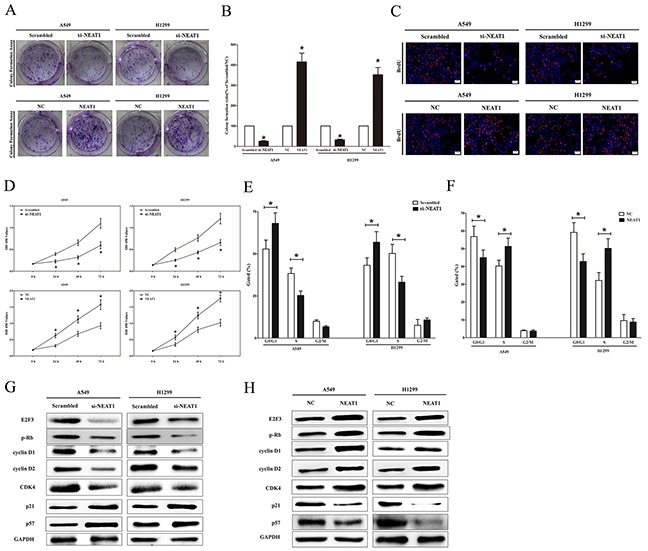
NEAT1 promotes tumor NSCLC cell growth in vitro **A.** Shown are representative photomicrographs of colony formation assay after transfection for fourteen days. **B.** Statistical analysis of colony formation assay. **C.** Shown are representative photomicrographs of BrdU staining in A549 and H1299 cells after transfection. Bar = 100 μm. **D.** CCK8 assays of A549 and H1299 cells after transfection. **F.** Cell-cycle analysis was performed after transfection for forty eight hours. The DNA content was quantified by flow cytometric analysis. **G.** Expression of E2F3, p-Rb, cyclin D1, cyclin D2, CDK4, p21 and p57 protein in transfected A549 and H1299 cells. Assays were performed in triplicate. *P < 0.05. Means ± SEM are shown. Statistical analysis was conducted using student t-test.

To further explore the oncogenic properties and roles of NEAT1 on NSCLC in vitro, we established NSCLC cell lines (A549 and H1299) with NEAT1 stable over-expression or transient knockdown (Using RNAi). Firstly, we evaluated the efficiency of NEAT1 on NSCLC cell growth. We used colony formation assay to assess NEAT1's role on clonogenic survival, and results demonstrated knockdown NEAT1 expression caused a decrease in the clonogenic survival of A549 and H1299 cells in comparison to that of in their counterparts (Figure 2A-2B). On the other hand, NEAT1 over-expressed cells (A549 and H1299) exhibited a significant increase in the clonogenic survival, in comparison to their counterparts (Figure 2A-2B). In addition, Our results of BrdU staining revealed that knockdown NEAT1 expression inhibited A549 and H1299 cell DNA synthesis by approximately 75% (Figure 2C) and 65% (Figure 2C), compared with blank A549 and blank H1299 cells, respectively. However, NEAT1 over-expressed A549 and H1299 cell DNA synthesis by approximately 2.3 folds (Figure 2C) and 1.9 folds (Figure 2C) compared with blank A549 and blank H1299 cells, respectively. To verify this result, we also did the CCK8 assay, and results demonstrated that knockdown of NEAT1 expression significantly attenuated A549 (52% of decrease) and H1299 (47% of decrease) cells vitality, while NEAT1 over-expression promoted A549 (1.7 folds of increase) and H1299 (2.1 folds of increase) cells vitality (Figure 2D). Furthermore, the growth inhibitory role of knockdown of NEAT1 on A549 and H1299 cells resulted in an increase in the proportion of cells in G1 and a decrease in the proportion of cells in the S phase (Figure 2E-2F).

We next examined the influence of NEAT1 on the expression of cyclin D1, a well-established human oncogene [44], which is over-expressed in lung cancer, breast cancer and pancreatic cancer [44–47], and over-expression of cyclin D1 is involved in malignant transformation in lung tissue [48]. Our results discovered that knockdown of NEAT1 expression remarkably decreased the protein expression of cyclin D1, while NEAT1 over-expression remarkably increased the level of cyclin D1 in A549 and H1299 cells (Figure 2G-2H). Cyclin D2 is highly expressed and promotes tumorigenesis in numerous of tumors [49, 50]. In our research, the protein expression of cyclin D2 was up-regulated by over-expression of NEAT1 (Figure 2G-2H). Our study revealed that the over-expression of NEAT1 is a mechanism for the down-regulation of p57 level in A549 and H1299 cells (Figure 2G-2H). Transfection of p21 (a cell cycle inhibitor) expressive constructs into normal [51] and tumor cell lines [52] leads to cell cycle arrest in G1 [53]. Our study revealed that NEAT1 down-regulated p21 level in A549 and H1299 cells (Figure 2G-2H). Our results also demonstrated that NEAT1 over-expression promoted protein levels of oncogenic E2F3 and CDK4 (Figure 2G and 2H).

Collectively, these results clearly revealed that NEAT1 markedly promoted cell growth in NSCLC cells.

### NEAT1 promotes NSCLC cell metastasis in vitro

**Figure 3:**
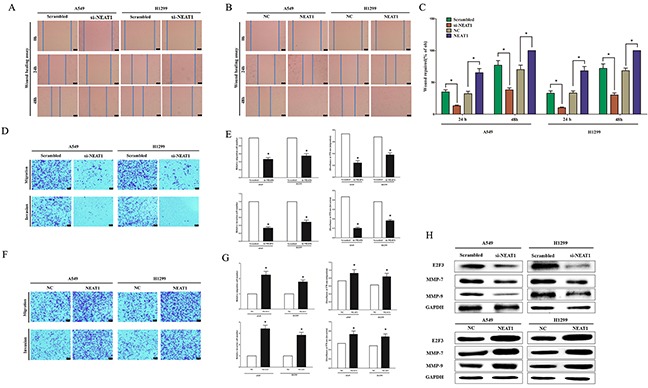
NEAT1 promotes NSCLC cell migration and invasion in vitro **A-B.** Shown are representative photomicrographs of “wound healing assay” in A549 and H1299 cells after transfection for 0 hour, twenty hours and forty eight hours. Bar = 50 μm. **C.** Statistical analysis of “wound healing assay”. **D-G.** A549 and H1299 cells were loaded onto the top well of a transwell inserts for cell migration or invasion assay. After twenty four hours, cells that migrated to the bottom chamber containing serum-supplemented medium were stained with 0.1% crystal violet, visualized under a phase-contrast microscope, and photographed. Bar = 50 μm. Total number of cells in five fields was counted manually. **H.** Expression of E2F3, MMP-7 and MMP-9 protein in A549 and H1299 cells after transfection. Assays were performed in triplicate. *P < 0.05. Means ± SEM are shown. Statistical analysis was conducted using student t-test.

To investigate whether the NEAT1 over-expression can promote NSCLC migration and invasion, we used two different approaches to evaluate the role of NEAT1 A549 and H1299 cells migration. In the first technique, we used a “scratch wound healing” assay. Motility of cells at different time points after generation of the wound was monitored under a microscope. Results demonstrated over expression of NEAT1 promoted migration in A549 and H1299 cells, while knock down of NEAT1 suppressed cell migration in A549 and H1299 cells (Figure 3A-3C). We also evaluated cancer cell migration and invasion through Transwell assays. Decreased NEAT1 expression impeded cell migration by 61% and 49% in A549 and H1299 cells, respectively (Figure 3D-3G), while NEAT1 over-expression promoted cell migration in A549 and H1299 cells. Similarly, A549 and H1299 cell invasion were also reduced by 85% and 91% after treating with si-NEAT1, respectively (Figure 3D-3G), while NEAT1 over-expression promoted cell invasion in A549 and H1299 cells.

We also explored the efficiency of NEAT1 on MMP-7 and MMP-9 expression, which all made sense in tumor metastasis, and results demonstrated silencing NEAT1 expression suppressed MMP-7 and MMP-9 protein expression both in A549 and H1299 cells (Figure 3H). As expected, NEAT1 over-expression significantly increased MMP-7 and MMP-9 protein expression both in A549 and H1299 cells (Figure 3H).

These results, taken together, clearly demonstrated that NEAT1 over-expression markedly promoted cell metastasis in NSCLC cells.

### NEAT1 inhibits NSCLC cell apoptosis

**Figure 4:**
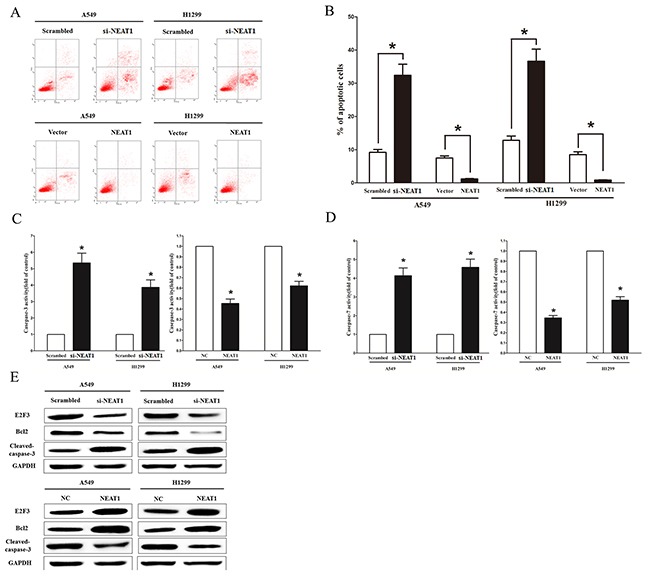
NEAT1 inhibits NSCLC cell apoptosis in vitro **A.** Shown are representative photomicrographs of flow cytometric analysis. **B.** Statistical analysis of flow cytometric analysis. **C-D.** Quantitative representation of caspase-3 and caspase-7 activity in A549 and H1299 cells after transfection for forty eight hours. **E.** Western-blot of E2F3, Bcl2 and cleaved-caspase-3 protein in A549 and H1299 cells after transfection. Assays were performed in triplicate. *P < 0.05. Means ± SEM are shown. Statistical analysis was conducted using student t-test.

We next investigate the role of NEAT1 on NSCLC cells apoptosis. Our results of flow cytometric analysis demonstrated that knockdown of NEAT1 expression resulted in a ~3.5-fold and ~3.7-fold increase in apoptotic cell death of A549 and H1299 cells (Figure 4A-4B), respectively. However, NEAT1 over-expression suppressed cell apoptosis in A549 and H1299 cells (Figure 4A-4B). In addition, we also assessed caspase-3 and caspase-7 activities after transfection of A549 and H1299 cells with pGCMV/NEAT1 or si-NEAT1, and results showed that knockdown of NEAT1 expression remarkably aggrandized the caspase-3 and caspase-7 activities in A549 and H1299 cell lysate than that of in their counterparts (Figure 4C-4D), respectively. Nevertheless, NEAT1 over-expression remarkably reduced the caspase-3 and caspase-7 activities in A549 and H1299 cell lysate, compared with that of in their counterparts (Figure 4C-4D), respectively. Moreover, NEAT1 over-expression also promoted the expression level of anti-apoptotic protein Bcl2 (Figure 4E), and suppressed the expression of cleaved-caspase-3 protein (Figure 4E) in A549 and H1299 cells. These results demonstrated that NEAT1 over-expression indeed suppressed apoptosis in A549 and H1299 cells.

### NEAT1 is a direct target of miR-377-3p

**Figure 5:**
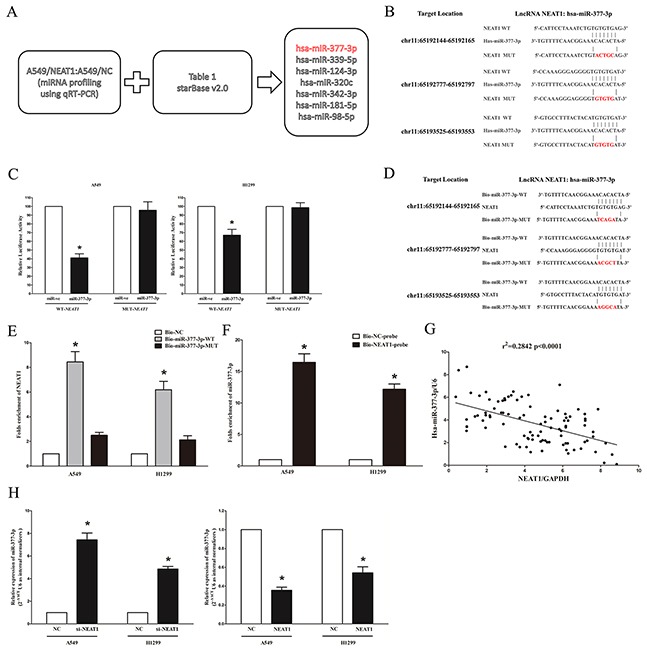
NEAT1 is a direct target of miR-377-3p **A.** Screen of the candidate miRNAs that interacted with NEAT1 by real-time PCR-based miRNA expression profiling and starBase (v2.0). Co-analysis of the down-regulated miRNAs in stable overexpression NEAT1/A549 cells compared to the control A549 cells and the miRNA list that potentially target NEAT1 predicted by starBase (v2.0), shown in Table 1, we got seven candidates. **B.** Sequence alignment of miR-377-3p with the putative binding sites within the wild-type regions of NEAT1. **C.** The luciferase report assay demonstrated that overexpression of miR-377-3p could reduce the intensity of fluorescence in A549 and H1299 cells transfected with the NEAT1-WT vector, while had no effect on the NEAT1-MUT vector. **D.**WT and the mutated forms of miR-377-3p sequence are shown. **E.** Detection of NEAT1 using qRT-PCR in the sample pulled down by biotinylated miR-377-3p. **F.** Detection of miR-377-3p using qRT-PCR in the sample pulled down by biotinylated NEAT1 probe. **G.** The correlation between NEAT1 mRNA and miR-377-3p expression in 96 lung cancer tissues. **H.** Detection of miR-377-3p using qRT-PCR in the si-NEAT1 or NEAT1 overexpression A549 and H1299 cell lines compared with control group. Assays were performed in triplicate. *P < 0.05. Means ± SEM are shown. Statistical analysis was conducted using student t-test.

Increasing publications have demonstrated that lncRNAs could act as a molecular sponge or a ceRNA in regulating the accumulation of miRNA and in turn affecting its biological functions. A database, starBase2.0 was used to predict and select miRNAs interacted with NEAT1 (results were shown in Table 2). Moreover, qRT-PCR-based miRNA profiling was used to select the differentially expressed miRNAs between NEAT1/A549 and A549/NC groups. Integrating the prediction by the bioinformatics and the verification by miRNA profiling analysis, miR-377-3p, miR-399-5p, miR-124-3p, miR-320c, miR-342-3p, miR-182-5p and miR-98-5p were selected as the candidate miRNAs (Figure 5A). Among these seven miRNAs, miR-377-3p (MIMAT0000730) was reduced the most at miRNA profiling analysis. Thus, it was chose to further study. Moreover, we also performed dual-luciferase reporter assay to further verify if NEAT1 was a functional target of miR-377-3p. Our results demonstratedmiR-377-3p may reduce the luciferase activity of pmirGLO-NEAT1-WT, while it may not affect the luciferase activity of pmirGLO-NEAT1-MUT (Figure 5C). Additionally, NEAT1 over-expression suppressed miR-377-3p expression both in A549 and H1299 cells (Figure 5D). Moreover, to find out whether miR-377-3p could pull down NEAT1, we applied a biotin-avidin pull-down assay. Results indicated NEAT1 was pulled down by miR-377-3p, but the introduction of mutations which disrupted the predicted miRNA recognition sites between NEAT1 and miR-377-3p results in the inability of miR-377-3p to pull down NEAT1 (Figures 5D and E), which indicated that the recognition of miR-377-3p to NEAT1 was in a sequence-specific manner. In addition, we also performed inverse pull-down assay using a biotin-labeled specific NEAT1 probe to verify whether NEAT1 could pull down miR-377-3p, and miR-377-3p was precipitated and analyzed by qRT-PCR analysis (Figure 5F). Furthermore, we assessed the association between NEAT1 mRNA and miR-377-3p expression in 96 NSCLC lung tissues as well, and results indicated the expression of NEAT1 mRNA and miR-377-3p showed a remarkably negative correlation as analyzed by Pearson correlation analysis (r^2^=0.2842, *P* <0.0001) (Figure 5G). Our results indicated that the knockdown of NEAT1 remarkably suppressed miR-377-3p expression (Figure 5H). These results revealed miR-377-3p could directly bind to NEAT1 at the miRNA recognition site.

**Table 2:** StarBase (v2.0) predicted the miRNAs that target NEAT1

name	mirAccession	geneName	targetSites	bioComplex	clipReadNum	cancerNum
hsa-miR-379-5p	MIMAT0000733	NEAT1	1	1	1975	8
hsa-let-7a-5p	MIMAT0000062	NEAT1	1	1	1975	6
hsa-let-7i-5p	MIMAT0000415	NEAT1	2	1	4721	6
hsa-miR-320d	MIMAT0006764	NEAT1	2	7	5	6
hsa-miR-433-3p	MIMAT0001627	NEAT1	1	6	0	6
hsa-miR-370-3p	MIMAT0000722	NEAT1	2	8	4137	6
hsa-miR-539-5p	MIMAT0003163	NEAT1	1	2	92	6
hsa-miR-125a-3p	MIMAT0004602	NEAT1	1	1	8	6
hsa-miR-28-5p	MIMAT0000085	NEAT1	1	1	12	6
hsa-let-7f-5p	MIMAT0000067	NEAT1	2	1	4721	6
hsa-miR-98-5p	MIMAT0000096	NEAT1	2	1	4721	6
hsa-miR-503-5p	MIMAT0002874	NEAT1	1	6	0	6
hsa-miR-214-3p	MIMAT0000271	NEAT1	1	1	12	5
hsa-miR-129-5p	MIMAT0000242	NEAT1	1	1	5	5
hsa-let-7e-5p	MIMAT0000066	NEAT1	2	1	4721	5
hsa-miR-216a-5p	MIMAT0000273	NEAT1	1	1	8	5
hsa-let-7c-5p	MIMAT0000064	NEAT1	1	1	1975	5
hsa-let-7b-5p	MIMAT0000063	NEAT1	1	1	1975	5
hsa-let-7g-5p	MIMAT0000414	NEAT1	2	1	4721	5
hsa-miR-335-5p	MIMAT0000765	NEAT1	1	6	0	5
hsa-let-7d-5p	MIMAT0000065	NEAT1	1	1	1975	5
hsa-miR-505-3p	MIMAT0002876	NEAT1	1	4	0	5
hsa-miR-107	MIMAT0000104	NEAT1	1	6	0	4
hsa-miR-495-3p	MIMAT0002817	NEAT1	1	7	1977	4
hsa-miR-324-5p	MIMAT0000761	NEAT1	2	6	0	4
hsa-miR-124-3p	MIMAT0000422	NEAT1	2	9	4522	4
hsa-miR-320b	MIMAT0005792	NEAT1	2	7	5	3
hsa-miR-146b-5p	MIMAT0002809	NEAT1	1	6	0	3
hsa-miR-329-3p	MIMAT0001629	NEAT1	2	7	5	3
hsa-miR-543	MIMAT0004954	NEAT1	1	2	0	3
hsa-miR-154-5p	MIMAT0000452	NEAT1	1	3	5	3
hsa-miR-365a-3p	MIMAT0000710	NEAT1	1	8	6328	3
hsa-miR-193a-3p	MIMAT0000459	NEAT1	1	7	2892	3
hsa-miR-10a-5p	MIMAT0000253	NEAT1	1	4	36	3
hsa-miR-27a-3p	MIMAT0000084	NEAT1	1	7	1722	3
hsa-miR-181d-5p	MIMAT0002821	NEAT1	2	8	4562	3
hsa-miR-10b-5p	MIMAT0000254	NEAT1	1	4	36	3
hsa-miR-499a-5p	MIMAT0002870	NEAT1	1	9	30	3
hsa-miR-320a	MIMAT0000510	NEAT1	2	7	5	3
hsa-miR-204-5p	MIMAT0000265	NEAT1	2	7	4397	3
hsa-miR-27b-3p	MIMAT0000419	NEAT1	1	7	1722	3
hsa-miR-504-5p	MIMAT0002875	NEAT1	1	6	0	3
hsa-miR-9-5p	MIMAT0000441	NEAT1	1	6	0	2
hsa-miR-181b-5p	MIMAT0000257	NEAT1	2	8	4562	2
hsa-miR-194-5p	MIMAT0000460	NEAT1	1	6	0	2
hsa-miR-708-5p	MIMAT0004926	NEAT1	1	1	12	2
hsa-miR-34c-5p	MIMAT0000686	NEAT1	1	1	1975	2
hsa-miR-342-3p	MIMAT0000753	NEAT1	2	8	9220	2
hsa-miR-377-3p	MIMAT0000730	NEAT1	3	8	8670	2
hsa-miR-193b-3p	MIMAT0002819	NEAT1	1	7	2892	2
hsa-miR-181c-5p	MIMAT0000258	NEAT1	2	8	4562	2
hsa-miR-125a-5p	MIMAT0000443	NEAT1	1	6	0	2
hsa-miR-103a-3p	MIMAT0000101	NEAT1	1	6	0	2
hsa-miR-3619-5p	MIMAT0017999	NEAT1	1	1	12	2
hsa-miR-146a-5p	MIMAT0000449	NEAT1	1	6	0	2
hsa-miR-339-5p	MIMAT0000764	NEAT1	3	6	0	2
hsa-miR-590-3p	MIMAT0004801	NEAT1	1	7	5	2
hsa-miR-383-5p	MIMAT0000738	NEAT1	1	1	5	2
hsa-miR-506-3p	MIMAT0002878	NEAT1	2	9	4522	2
hsa-miR-34a-5p	MIMAT0000255	NEAT1	1	1	1975	1
hsa-miR-101-3p	MIMAT0000099	NEAT1	1	1	14	1
hsa-miR-181a-5p	MIMAT0000256	NEAT1	2	8	4562	1
hsa-miR-202-3p	MIMAT0002811	NEAT1	1	1	5	1
hsa-miR-211-5p	MIMAT0000268	NEAT1	2	7	4397	1
hsa-miR-22-3p	MIMAT0000077	NEAT1	1	1	5	1
hsa-miR-320c	MIMAT0005793	NEAT1	2	7	5	1
hsa-miR-371a-5p	MIMAT0004687	NEAT1	1	4	86	1
hsa-miR-3139	MIMAT0015007	NEAT1	1	1	12	1
hsa-miR-449a	MIMAT0001541	NEAT1	1	1	1975	1
hsa-miR-362-3p	MIMAT0004683	NEAT1	2	7	5	1
hsa-miR-761	MIMAT0010364	NEAT1	1	1	12	0
hsa-miR-425-5p	MIMAT0003393	NEAT1	1	8	6328	0
hsa-miR-449b-5p	MIMAT0003327	NEAT1	1	1	1975	0
hsa-miR-4500	MIMAT0019036	NEAT1	1	1	1975	−1
hsa-miR-3529-5p	MIMAT0019828	NEAT1	1	1	1975	−1
hsa-miR-4725-5p	MIMAT0019843	NEAT1	1	6	0	−1
hsa-miR-4319	MIMAT0016870	NEAT1	1	6	0	−1
hsa-miR-4429	MIMAT0018944	NEAT1	2	7	5	−1
hsa-miR-4262	MIMAT0016894	NEAT1	2	8	4562	−1
hsa-miR-4458	MIMAT0018980	NEAT1	1	1	1975	−1

### NEAT1's oncogenic functions are partially through negative regulation of miRNA-377-3p

**Figure 6:**
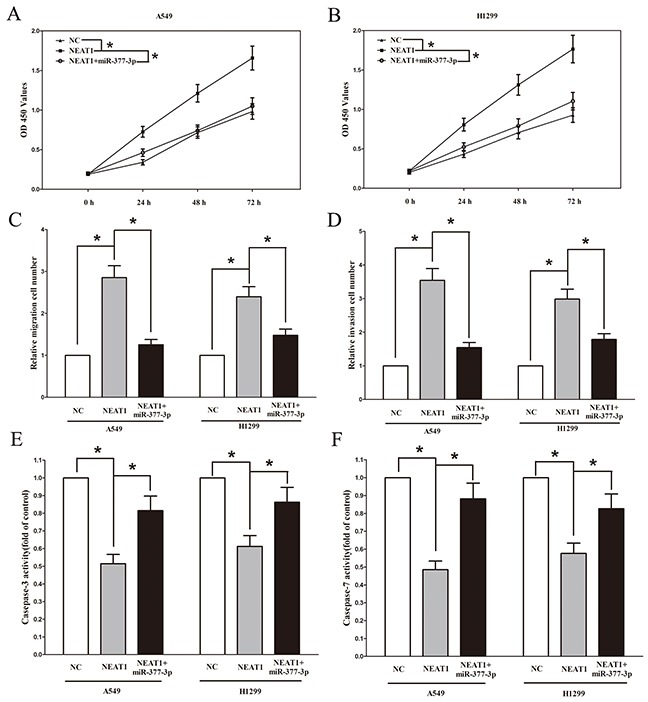
NEAT1's oncogenic activity is in part through negative regulation of miRNA-377-3p in NSCLC cells Up-regulated miR-377-3p in A549 and H1299 cells, which stably overexpressed NEAT1, largely reversed the favorable effects of NEAT1 on cell proliferation **A-B.** migration **C.** and invasion **D.** Moreover, overexpression of miR-377-3p largely increased the cell apoptosis inhibited by NEAT1 **E-F.** Assays were performed in triplicate. *P < 0.05. Means ± SEM are shown. Statistical analysis was conducted using student One-Way ANOVA test.

Having verified NEAT1 was a direct target of miR-377-3p, the mechanism of miR-377-3p in NEAT1-induced inhibition on NSCLC cells was still unclear. Up-regulated miR-377-3p in A549 and H1299 cells, which stably over-expressed NEAT1, significantly reversed the favorable roles of NEAT1 on cell growth and metastasis, in NSCLC cells (Figure 6A-6D). In addition, miR-377-3p over-expression significantly promotes cell apoptosis inhibited by over-expressed-NEAT1 treatment (Figure 6E and 6F). These results confirmed that miR-377-3p made sense in NEAT1-induced inhibitory roles on NSCLC cells.

### MiR-377-3p directly targets oncogene E2F3

**Figure 7:**
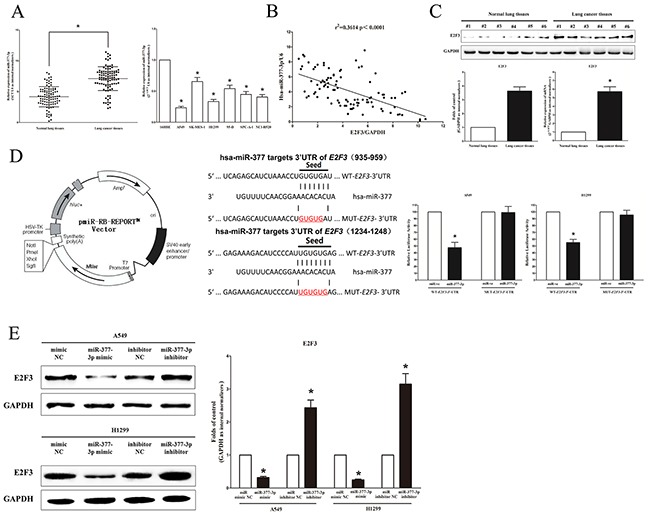
E2F3 proto-oncogene is a target of miR-377-3p at specific 3′-UTR sites **A.** miR-377-3p is lowly expressed in primary human lung cancer tissues (n=96) and NSCLC cell lines. **B.** Scatter plots showing the inverse association between miR-377-3p level and E2F3 mRNA expression in primary human lung cancer tissues (n=96). **C.** E2F3 is up-regulated in primary human lung cancer tissues (n=96). **D. Left** pmiR-RB-REPORT™ dual-luciferase reporter vector. **Middle** The 3′-UTR of E2F3 harbors two miR-377-3p cognate sites. **Right** Relative luciferase activity of reporter plasmids carrying wild-type or mutant E2F3 3′-UTR in A549 and H1299 cells co-transfected with negative control (NC) or miR-377-3p mimic. **E.** miR-377-3p suppressed the protein expression of E2F3. Assays were performed in triplicate. *P < 0.05. Means ± SEM are shown. Statistical analysis was conducted using student t-test.

Having confirmed NEAT1 could reversely regulate miR-377-3p expression, we then investigate its functional roles. Firstly, we examined miR-377-3p expression in NSCLC lung tissues and their pair-matched adjacent normal lung tissues, and in NSCLC cell lines (including A549, 95D, H1299, SPC-A-1, SK-MES-1, and NCI-H520 cells), and results revealed that of miR-377-3p expression was remarkably suppressed (mean=29% of decrease) in 96 NSCLC tissues compared with their 96 pair-matched adjacent normal lung tissues (Figure 7A). We also found a lower expression of miR-377-3p in A549, SK-MES-1, 95D, H1299, SPC-A-1 and NCI-H520 cells, in comparison to that of 16HBE cells (Figure 7A). Furthermore, we also assessed the association between the expression of E2F3 mRNA and miR-377-3p in 96 NSCLC lung tissues. And results indicated expression of E2F3 mRNA and miR-377-3p showed a remarkably negative correlation as analyzed by Pearson correlation analysis (r^2^=0.3614, *P* <0.0001) (Figure 7B). Additionally, we examined E2F3 expression in NSCLC lung tissues and pair-matched adjacent normal lung tissues, and results indicated the expression of E2F3 protein was over-expressed in NSCLC lung tissues in comparison to pair-matched adjacent normal lung tissues (Figure 7C). These results were verified by E2F3 mRNA expression using qRT-PCR assay (Figure 7C). To investigate the effect of miR-377-3p on NSCLC, we screen Targetscan, miRanda, PicTar to select potential predicted targets of miR-377-3p. We identified the top 100 potential targets, and among them, we found a distinguished oncogene, E2F3, which was up-regulated in numerous of malignancies. These findings indicated that E2F3 could be a direct target of miR-377-3p in NSCLC. Next, we used luciferase reporter assays to determine whether E2F3 expression were indeed regulated by miR-377-3p, and results demonstrated that miR-377-3p inhibited luciferase activity in A549 cells and H1299 cells when the reporter plasmid carried a WT E2F3 3′-UTR, but no significant suppression was observed at the reporter plasmid carried a mutant E2F3 3′-UTR (Figure 7D). Next, we explored the effect of miR-377-3p on the protein and mRNA expression of E2F3. Our results revealed that miR--377-3p suppressed the expression of E2F3 protein and mRNA, in comparison to their counterparts (Figure 7E), respectively. Our results reveal that miR-377-3p targets human E2F3 by directly binding to the predicted sites in 3′-UTR of E2F3 mRNA.

### MiR-377-3p exerts tumor suppressive function through down-regulation of E2F3

**Figure 8:**
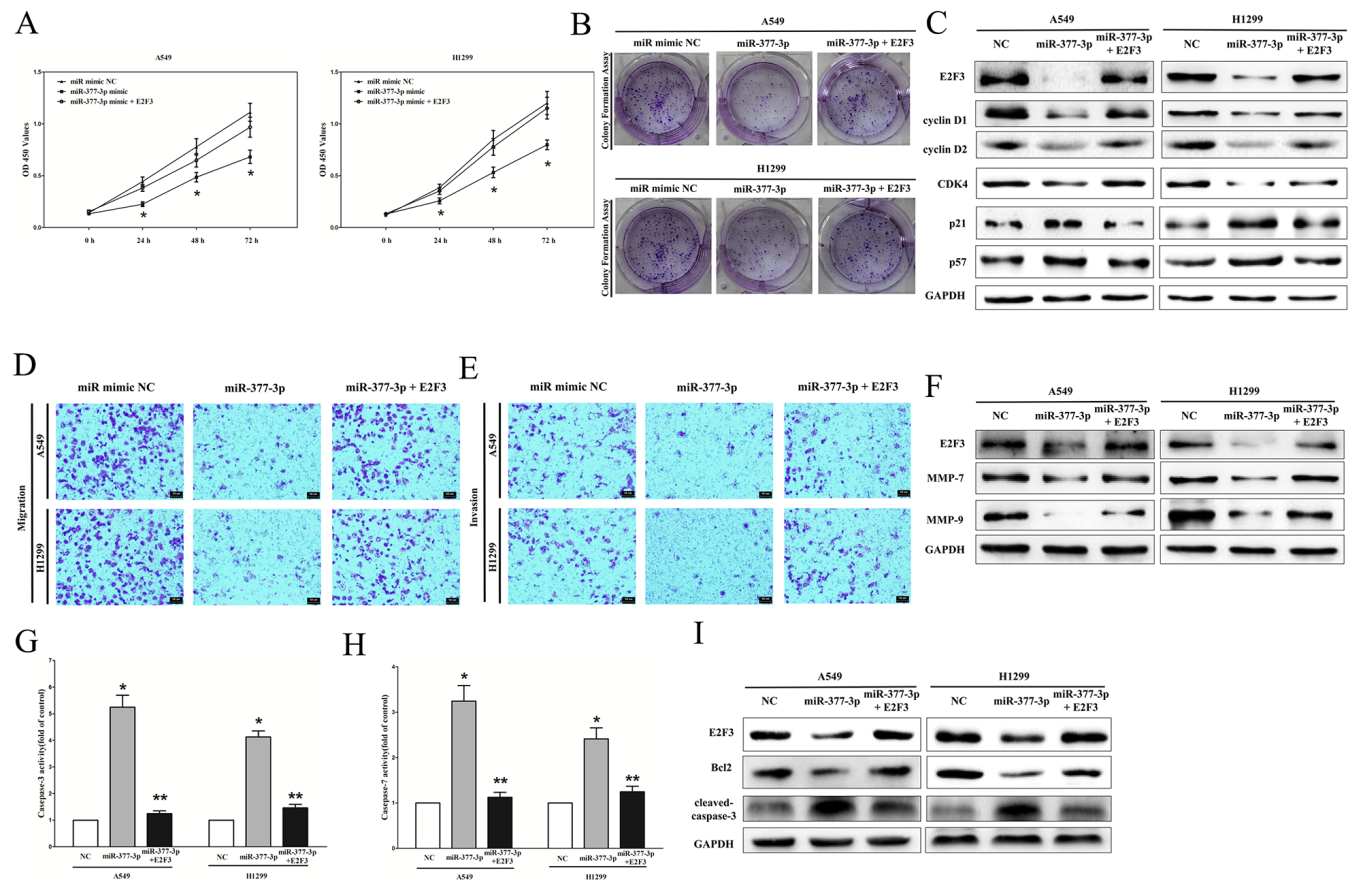
miR-377-3p exerts tumor suppressor function through down-regulation of E2F3 in NSCLC cell lines **A-B.** CCK8 and colony formation assays demonstrated E2F3 reversed the growth inhibitory role of miR-377-3p in A549 and H1299 cells. **C.** Protein expression of E2F3, cyclin D1, cyclin D2, CDK4, p21, and p57 after transfection. **D-E.** Transwell migration/invasion assays demonstrated that E2F3 reversed the inhibitory role of miR-377-3p on migration and invasion in A549 and H1299 cells. **F.** Protein expression of E2F3, MMP-7, and MMP-9 after transfection. **G-H.** Caspase-3 and caspase-7 activity assays demonstrated that E2F3 reversed the favorable role of miR-377-3p on apoptosis in A549 and H1299 cells. **I.** Protein expression of E2F3, Bcl2, and cleaved-caspase-3 after transfection. Assays were performed in triplicate. *P < 0.05, **P < 0.05. Means ± SEM are shown. Statistical analysis was conducted using student One-Way ANOVA test.

Next, we investigated the mechanism of miR-377-3p on progression in NSCLC cells. Firstly, we examined its effects on NSCLC cell growth. CCK8 assays revealed that miR-377-3p significantly reduced A549 and H1299 cells vitality, while when co-transfected pGCMV/E2F3 and miR-377-3p in A549 and H1299 cells, cells vitality was remarkably increased and normalized to that of in their counterparts (Figure 8A). In addition, colony formation assay also revealed that E2F3 over-expression reversed the growth-inhibitory efficiency of miR-377-3p in A549 and H1299 cells (Figure 8B). Further, western-blot demonstrated the miR-377-3p suppressed the protein expression of E2F3, cyclin D1, cyclin D2 and CDK4, and increased the protein expression of p21 and p57 in A549 and H1299 cells, while E2F3 over-expression reversed the favorable efficiency of miR-377-3p on up-regulation of protein levels of E2F3, cyclin D1, cyclin D2 and CDK4, and reversed the inhibitory efficiency of miR-377-3p on the protein levels of p21 and p57 in A549 and H1299 cells (Figure 8C). These data demonstrated miR-377-3p suppressed NSCLC cell growth by targeting E2F3.

Next, we investigated the effect of miR-377-3p on NSCLC cell metastasis. Our results of Transwell migration/invasion assays demonstrated miR-377-3p inhibited A549 and H1299 cell metastasis through inhibition of E2F3 (Figure 8D-8E). We also discovered miR-377-3p suppressed MMP-7 and MMP-9 protein levels in A549 and H1299 cells, and E2F3 reversed the inhibitory effect of miR-377-3p on MMP-7 and MMP-9 protein expression (Figure 8F). These results indicated miR-377-3p inhibited NSCLC cell metastasis by targeting E2F3.

We then evaluated the effect of miR-377-3p on NSCLC cell apoptosis. Firstly, we examined the caspase-3/7 activities in A549 and H1299 cells after trasfection, and results indicated that miR-377-3p remarkably aggrandized the caspase-3/7 activities in miR-377-3p treated A549 and H1299 cell lysate, compared with that of in their counterparts (Figure 8G-8H), respectively. While E2F3 over-expression remarkably reversed the favorable effect of miR-377-3p on reduction of the caspase-3/7 activities in A549 and H1299 cell lysate (Figure 8G-8H). Moreover, over-expression of E2F3 also reversed the suppressive role of miR-377-3p on the expression level of anti-apoptotic protein Bcl2 (Figure 8I), and reversed the favorable effect of miR-377-3p on promotion of cleaved-caspase-3 protein expression (Figure 8I). Our results confirmed that miR-377-3p promoted apoptosis in NSCLC cells through down-regulation of E2F3.

### NEAT1 promotes NSCLC cell growth in vivo by inhibiting miR-377-3p/E2F3 axis

**Figure 9:**
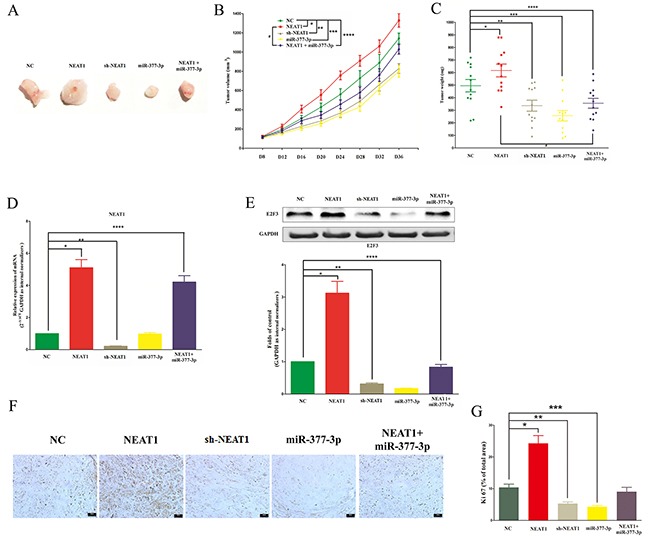
NEAT1 promotes NSCLC cell growth in vivo by inhibiting miR-377-3p/E2F3 axis **A-C.** Tumor size, volume, and weight of subcutaneous implantation models of A549 cell are shown. **D.** NEAT1 expression in tumors isolated from NC, NEAT1, sh-NEAT1, miR-377-3p, and NEAT1+miR-377-3p groups. **E.** The protein expression of E2F3 in tumors isolated from NC, NEAT1, sh-NEAT1, miR-377-3p, and NEAT1+miR-377-3p groups. **F.** Immunohistochemistry of Ki67 in tumors isolated from NC, NEAT1, sh-NEAT1, miR-377-3p, and NEAT1+miR-377-3p groups. **G.** Statistics of Ki 67 IHC. Assays were performed in triplicate. *P < 0.05, **P < 0.05, ***P < 0.05, ****P < 0.05. Means ± SEM are shown. Statistical analysis was conducted using student One-Way ANOVA test.

To verify the effects of NEAT1 on tumorigenesis in vivo, pGCMV/sh-NEAT1 cells, pGCMV/NEAT1 cells or appropriate control cells were subcutaneously injected into nude mice. 5 nmol miR-377-3p agomir was directly injected into the implanted tumor at the eighth day after injection every four day. Knockdown NEAT1 significantly decreased tumor growth in vivo compared with negative controls (NC) (Figure 9A-9C). In contrast, xenograft tumors from pGCMV/NEAT1 cells grew significantly faster than the tumors from control cells (NC) (Figure 9A-9C). Moreover, miR-377-3p also significantly decreased tumor growth in vivo compared with negative controls (NC) (Figure 9A-9C). And miR-377-3p treated xenograft tumors from pGCMV/NEAT1 cells (Figure 5A, B) showed decreased tumor growth in vivo compared with xenograft tumors from pGCMV/NEAT1 cells (Figure 9A-9C). Using qRT-PCR and in situ hybridisation analysis, we confirmed the NEAT1 knockdown or over-expression in the xenograft tumors generated from pGCMV/sh-NEAT1 cells or pGCMV/NEAT1 cells, respectively (Figure 9D). Moreover, western-blot of E2F3 demonstrated that up-regulation of NEAT1 promoted the protein expression of E2F3 (Figure 9E), and down-regulation of NEAT1 inhibited the protein expression of E2F3 (Figure 9E), miR-377-3p suppressed the protein expression of E2F3 (Figure 9E), and miR-377-3p treatment decreased the high protein levels of E2F3 in xenograft tumors generated from pGCMV/NEAT1 cells (treated with miR-377-3p agomir every four day) (Figure 9E).

To further investigate the in vivo effects of NEAT1, tumor cell proliferation was assessed using proliferation-related nuclear antigen Ki67 immunoreactivity assay. As shown in Figure 9F and G, up-regulation of NEAT1 promoted tumor cell proliferation, and down-regulation of NEAT1 inhibited tumor cell proliferation. Taken together, these results demonstrate that NEAT1 plays a crucial role on NSCLC progression through inhibition of miR-377-3p/E2F3 axis.

## DISCUSSION

Recent researches have revealed the functional roles of lncRNAs [54–56], and provided insights into the molecular mechanisms by which lncRNAs function in a variety of human tumors [57–65]. Nevertheless, the mechanisms of NEAT1 in NSCLC have not been thoroughly elaborated. Our present study provided new evidence that highly over-expressed NEAT1 in NSCLC lung tissues and cell lines acted an oncogenic role. NEAT1 was revealed as a direct target of miR-377-3p and there was an interactive suppression between them. NEAT1 acts as an oncogene to promote tumor progression in NSCLC in large part attributed to its ability to inhibit miR-377-3p (acting as “competitive endogenous RNAs (ceRNAs)”) and subsequent activation of the E2F3 signaling pathway. Thus, our study contributes to an increasing of literatures supporting the importance of non-annotated lncRNAs species in the field of cancer research.

In this study, we found NEAT1 expression in NSCLC lung tissues was remarkably higher than that of in adjacent normal lung tissues. Specifically, NEAT1 expression was remarkably higher in larger tumors and at later stages of tumor development. Our study also demonstrated a correction between NEAT1 expression levels and NSCLC prognosis or therapeutic outcome. A strong correction of high NEAT1 expression in tumors with poor survival was confirmed in 96 NSCLC samples. The correction was independent from other clinical covariates, revealing that NEAT1 expression levels could be as a useful prognostic biomarker to help identify patients who are at a higher risk of NSCLC progression, which were in accordance with Pan and his colleagues’ findings [66]. In addition, NEAT1 significantly increased NSCLC cell viability, G1–G0 phase arrest, migration and invasion, and inhibited cell apoptosis in vitro, while NEAT1 knockdown reversed it. Collectively, our observations demonstrate NEAT1 may serve as an oncogene and could play a critical efficiency in NSCLC initial, development and progression.

Although NEAT1 has been suggested to act as an oncogene, the underlying mechanism by which NEAT1-mediated gene expression participates in tumorigenesis remains to be clarified. NEAT1 plays roles on controlling numerous of biological processes, such as cellular differentiation and stress response through paraspeckles pathway [39–41]. Recently, Hirose et al. evaluated the effect of NEAT1 on transcriptional regulation by sequestering SFPQ from the RNA-specific adenosine deaminase B2 (ADARB2) gene in response to proteasome inhibition [67, 68]. In our present study, we aimed to discover another underlying molecular mechanism of NEAT1 on NSCLC progression, namely, functioning as “molecular sponges” to regulate microRNAs. It was reported that LncRNAs played a crucial effect in multiple processes in cells through acting as ceRNAs to regulate microRNAs [67]. Numerous of lncRNAs have been evaluated, including lncRNA GAS5 [68, 69], and CCAT1 [70]. In our study, we investigated the effect of NEAT1 on NSCLC cells and discovered that NEAT1 involved in the ceRNA regulatory network and functioned as endogenous miRNA sponges to bind to miR-377-3p and regulated its function [71]. Recent studies indicated miR-377 showed tumor suppressive role on malignant melanoma [72], human clear cell renal cell carcinoma (CCRCC) [73], and hepatocellular carcinoma [74], while its role on NSCLC had not been investigated. In this study, we found miR-377-3p was down-expressed in NSCLC lung tissues and cell lines, and miR-377-3p suppressed cell growth, metastasis, and induced cell apoptosis in A549 and H1299 cells. In addition, our results also revealed the expression of NEAT1 and miR-377-3p showed a significantly negative correlation in NSCLC tissues. And knockdown of NEAT1 remarkably increased miR-377-3p expression, while over-expression of NEAT1 decreased its expression level. Moreover, biotin-avidin pull-down system demonstrated NEAT1 could pull down miR-377-3p. In addition, our study also demonstrated that miR-377-3p could reverse the favorable effects of NEAT1 on cell growth, metastasis and negative role of NEAT1 on cell apoptosis in NSCLC cell lines, which demonstrated NEAT1 played its favorable role on NSCLC progression, at least in part, through inhibition of miR-377-3p.

Having shown the critical effect of miR-377-3p on suppressing NSCLC progression, we searched for the potential gene effectors involved in its function. MiR-377 can regulate numerous of target genes. Recent study indicated that miR-377 targeted oncogenic ETS1 gene and functioned as a tumor suppressor in CCRCC [75], and miR-377 inhibited TIAM1 expression and repressed cell growth and metastasis in hepatocellular carcinoma [76]. But among all of the predicted target genes of miR-377-3p, we discovered E2F3 acted as a crucial effector of miR-377-3p. Recent research demonstrated the G1/S transition of the cell cycle was partly controled by E2F transcription factors [77, 78]. E2F family control genes expression which participated in numerous of cellular process, such as differentiation, mitosis, and apoptosis [78–80]. E2F3 is not only required in normal proliferation, but also the key regulator on limiting the proliferation of tumor cell lines [81]. Dyregulation of E2Fs expression have been interacted with a variety of types of cancers, and E2F3 has been found to be over-expressed in bladder, lung and prostate cancers [78, 82–85]. In our study, highly expression of E2F3 expression was found in NSCLC than pair-matched adjacent lung tissues. Using bioinformatics, we verified E2F3 as a direct target of miR-377-3p, supporting by luciferase reporter assays that miR-377-3p targeted E2F3 mRNA at its 3′-UTR. Moreover, our results also demonstrated miR-377-3p exerted its tumor suppressive role on NSCLC through targeting E2F3. Additionally, tumor formation in BALB/c nude mice confirmed NEAT1 promoted NSCLC cell growth in vivo by inhibiting miR-377-3p/E2F3 axis

Taken together, our results indicate that highly expressed NEAT1 is an oncogenic lncRNA that promotes the tumorigenesis and progression of NSCLC through miR-377-3p-E2F3 axis. NEAT1 may also act as a prognostic factor in NSCLC. The present results elucidate a potential mechanism underlying the tumor-oncogenic role of NEAT1 in NSCLC, and indicate that NEAT1 could be a useful marker and potential therapeutic target in NSCLC.

## MATERIALS AND METHODS

### Tissue collection

Lung cancer tissues and normal tissues were obtained from patients who had undergone surgery at the People's Hospital of Wuhan University, between 2011 and 2015 and who were diagnosed with lung cancer based on histopathological evaluation. No local or systemic treatment had been conducted in these patients before the operation. All the tissue samples were collected, immediately snap frozen in liquid nitrogen, and stored at −80°C until RNA extraction. The study was approved by the Research Ethics Committee of Wuhan University (Wuhan, Hubei, PR China). Informed consent was obtained from all patients.

### Cell lines and culture conditions

Five NSCLC cancer cell lines (A549, SPC-A1, H1299, 95D, SK-MES-1, and NCI-H520) and human bronchial epithelial (16HBE) cell were purchased from the Institute of Biochemistry and Cell Biology of the Chinese Academy of Sciences (Shanghai, China). Cells were maintained in RPMI 1640 (GIBCO-BRL; Invitrogen, Carlsbad, CA) supplemented with 10% fetal bovine serum (FBS) and antibiotics and cultured at 37°C in humidified air with 5% CO2.

### Transfection of cell lines

The siRNA (small interfering RNA) sequences were as follows: NEAT1 (NR_131012) siRNA1 (5′-GCCAUCAGCUUUGAAUAAAUU-3′), NEAT1 siRNA2 (5′-GGUGUUAUCAAGUGAAUUAUU-3′), NEAT1 siRNA3 (5′-GCCUUGUAAAUGCCU AUAUUU-3′). Synthetic sequence-scrambled siRNA from Invitrogen were used as negative controls. Hsa-miRNA-377-3p mimic and mimic negative control, hsa-miRNA-377-3p inhibitor and inhibitor negative control were purchased from RiboBio Co., Ltd. (Guangzhou, China). For convenience, hsa-miRNA-377-3p mimic and mimic negative control, hsa-miRNA-377-3p inhibitor and inhibitor negative control were simply referred to as miR-377-3p mimic and miR mimic NC, miR-377-3p inhibitor and miR inhibitor NC, respectively. The target sequence of shRNA-NEAT1 was as follows: GCCATCAGCTTTGAATAAATT. Human NEAT1 gene (NR_131012) and E2F3 gene (NM_001949) was ligated into pGCMV/MCS/RFP/Neo vector (GenePharma, Shanghai, China). The empty vector was used as a negative control (NC). Stable cell lines were created by selection with Geneticin (G418; Invitrogen, CA, USA). The NEAT1 and control siRNAs, miR-377-3p mimic and miR mimic NC, miR-377-3p inhibitor and miR inhibitor NC, shRNA-NEAT1, pGCMV/NEAT1 and NC, pGCMV/E2F3 and NC were transfected into A549 and H1299 cells at approximately 50%–70% confluence, which were cultured on six-well plates using Opti-MEM I and Lipofectamine 2000 (Invitrogen, CA, USA) according to the manufacturer's protocol.

### Western blot analysis

Forty eight hours after transfection, total protein was extracted from the A549 and H1299 cells using RIPA cell lysis reagent containing proteinase and phosphatase inhibitors (Solarbio) at 4°C for 30 min [86–90]. Cell lysates were centrifuged at 12,000 × g for 20 min at 4°C, and the protein concentrations of the supernatant were determined using the BCA protein assay reagent kit (Thermo). The supernatants containing total protein were then mixed with a corresponding volume of 5 × SDS loading buffer and heated at 100°C for 10 min. Then, the supernatant lysates were run on 10% SDS-polyacrylamide gels (50 μg/lane), and proteins were transferred to poly (vinylidene fluoride) (PVDF) membranes (Hertfordshire, UK) by semidry electroblotting (1.5 mA/cm2). PVDF membranes were then incubated in blocking buffer [Tris-buffered saline (TBS) supplemented with 0.05% (vol/vol) Tween 20; TBST] containing 5% (wt/vol) skimmed milk powder for 120 min at room temperature followed by three 10 min washes in TBST. The PVDF membranes were then incubated with anti-E2F3 (1:1000 dilutions, Affinity), anti-Bcl2 (1:1,000 dilutions, Affinity), anti-caspase3 (1:1,000 dilutions, Affinity), anti-cyclin D1 (1:1,000 dilutions, Affinity), anti-cyclin D2 (1:1,000 dilutions, Affinity), anti-CDK4 (1:1,000 dilutions, Affinity), anti-CDK6 (1:1,000 dilutions, Affinity), anti-MMP7 (1:1,000 dilutions, Affinity), anti-MMP9 (1:1,000 dilutions, Affinity) and anti-GADPH (1:5,000 dilutions, Affinity) as internal normalizers in TBST containing 5% (wt/vol) skimmed milk powder (antibody buffer) overnight at 4°C on a three-dimensional rocking table. Then the membranes were washed three times for 10 min in TBST and then incubated with goat anti-rabbit IgG conjugated to horseradish peroxidase (1:12,000 dilutions) in antibody buffer for 120 min. Finally, membranes were washed three times for 10 min in TBST and exposed to ECL Advance reagent (GE Healthcare Biosciences, Buckinghamshire, UK) for 2 min as described in the manufacturer's protocol. Then membranes were exposed to Hyperfilm-ECL (GE Healthcare Bio-Sciences) for 2–5 min and visualized using a Fluor S Multimager and Quantity One 4.1 (Bio-Rad Laboratories, Hercules, CA). The molecular weights of the bands were calculated by a comparison with prestained molecular weight markers (molecular weight range: 6,500 −250,000) that were run in parallel with the samples. Semiquantitative analysis of specific immunolabeled bands was performed using a Fluor S image analyzer and Quantity One 4.1.

### RNA isolation and quantitative reverse transcription poly-merase chain reaction (qRT-PCR)

Total RNA from the cultured cells was extracted using Trizol reagent (Invitrogen) according to the manufacturer's instructions. MiRNA levels were measured by qRT-PCR. RNA (2 μg) was converted into cDNA using the miDETECT A Track™ miRNA qRT-PCR Starter Kit (RiboBio, Guangzhou, China) according to the manufacturer's instructions. Premiers were shown in Table 3. QRT-PCR was performed using SYBR^®^ Premix Ex Taq™ II (Takara) in the ABI PRISM® 7300 real-time PCR system (Applied Biosystems, Foster City, CA, USA). GAPDH and U6 were used as endogenous controls. In addition, melting curves were used to evaluate non-specific amplification. The relative expression level was calculated using the 2^−ΔΔCt^ method. The primer sequences used in this study are as follows: GAPDH: forward 5′-TGCACCACCAACTGCTTAGC-3′, reverse 5′-GGCATGCACTGTGGTCATGAG-3′, miR-377-3p: forward 5′-GGGAGGCAGTGTATTGTTA-3′, reverse 5′-CAGTGCGTGTCGTGGAGT-3′, NEAT1_1: forward 5-CTTCCTCCCTTTAACTTATCCATTCAC-3′, reverse 5′-CTCTTCCTCCACCATTACCAACAATAC-3′, NEAT1_2: forward 5′-CAGTTAGTTTATCAGTTCTCCCATCCA-3′, reverse 5′-GTTGTTGTCGTCACCTTTCAACTCT-3′, E2F3: forward 5′-AGCGATTGCTCAGTTTCTAT-3′, reverse 5′-GTTCACACACGGTCCTTCTA-3′. The for- mula and its derivations were obtained from the ABI Prism 7300 sequence detection system user guide. Statistical analysis was performed on the fold change.

**Table 3:** Primer sequences for quantitative reverse transcription(RT)-PCR (miRNA)

Name	mirAccession	sequence
hsa-miR-379-5p	MIMAT0000733	RT-premier:5′-GTCGTATCCAGTGCAGGGTCCGAGGTATTCGCACTGGATACGACCCTACG-3′ sense:5′-ACGGGCTGGTAGACTATGGCAC-3′ antisense:5′-CGCAGGGTCCGAGGTATTC-3′
hsa-let-7a-5p	MIMAT0000062	RT-premier:5′-GTCGTATCCAGTGCAGGGTCCGAGGTATTCGCACTGGATACGACAACTAT-3′ sense:5′-ACGGGCTGAGGTAGTAGGTTGT-3′ antisense:5′-CGCAGGGTCCGAGGTATTC-3′
hsa-let-7i-5p	MIMAT0000415	RT-premier:5′-GTCGTATCCAGTGCAGGGTCCGAGGTATTCGCACTGGATACGACAACAGC-3′ sense:5′-ACGGGCTGAGGTAGTAGTTTGT-3′ antisense:5′-CGCAGGGTCCGAGGTATTC-3′
hsa-miR-320d	MIMAT0006764	RT-premier:5′-GTCGTATCCAGTGCAGGGTCCGAGGTATTCGCACTGGATACGACTCCTCT-3′ sense:5′-ACGGGTAAAAACTGGGTTGAGA-3′ antisense:5′-CGCAGGGTCCGAGGTATTC-3′
hsa-miR-433-3p	MIMAT0001627	RT-premier:5′-GTCGTATCCAGTGCAGGGTCCGAGGTATTCGCACTGGATACGACACACCG-3′ sense:5′-ACGGGTATCTTGATGGGCTTCT-3′ antisense:5′-CGCAGGGTCCGAGGTATTC-3′
hsa-miR-370-3p	MIMAT0000722	RT-premier:5′-GTCGTATCCAGTGCAGGGTCCGAGGTATTCGCACTGGATACGACACCAGG-3′ sense:5′-AGCCGAGCCCGCTGGGGTGTAA-3′ antisense:5′-CGCAGGGTCCGAGGTATTC-3′
hsa-miR-539-5p	MIMAT0003163	RT-premier:5′-GTCGTATCCAGTGCAGGGTCCGAGGTATTCGCACTGGATACGACACACAC-3′ sense:5′-ACGGGCGGCGAACTTATCCTTG-3′ antisense:5′-CGCAGGGTCCGAGGTATTC-3′
hsa-miR-125a-3p	MIMAT0004602	RT-premier:5′-GTCGTATCCAGTGCAGGGTCCGAGGTATTCGCACTGGATACGACGGCTCC-3′ sense:5′-ACGGGCACAGGTGAGGTTCTTG-3′ antisense:5′-CGCAGGGTCCGAGGTATTC-3′
hsa-miR-28-5p	MIMAT0000085	RT-premier:5′-GTCGTATCCAGTGCAGGGTCCGAGGTATTCGCACTGGATACGACCTCAAT-3′ sense:5′-ACGGGCAAGGAGGTCACAGTCT-3′ antisense:5′-CGCAGGGTCCGAGGTATTC-3′
hsa-let-7f-5p	MIMAT0000067	RT-premier:5′-GTCGTATCCAGTGCAGGGTCCGAGGTATTCGCACTGGATACGACAACTAT-3′ sense:5′-ACGGGCTGAGGTAGTAGATTGT-3′ antisense:5′-CGCAGGGTCCGAGGTATTC-3′
hsa-miR-98-5p	MIMAT0000096	RT-premier:5′-GTCGTATCCAGTGCAGGGTCCGAGGTATTCGCACTGGATACGACAACAAT-3′ sense:5′-ACGGGCTGAGGTAGTAAGTTGT-3′ antisense:5′-CGCAGGGTCCGAGGTATTC-3′
hsa-miR-503-5p	MIMAT0002874	RT-premier:5′-GTCGTATCCAGTGCAGGGTCCGAGGTATTCGCACTGGATACGACCTGCAG-3′ sense:5′-ACGGGGTAGCAGCGGGCACAGT-3′ antisense:5′-CGCAGGGTCCGAGGTATTC-3′
hsa-miR-214-3p	MIMAT0000271	RT-premier:5′-GTCGTATCCAGTGCAGGGTCCGAGGTATTCGCACTGGATACGACACTGCC-3′ sense:5′-ACGGGCACAGCAGGCACAGACA-3′ antisense:5′-CGCAGGGTCCGAGGTATTC-3′
hsa-miR-129-5p	MIMAT0000242	RT-premier:5′-GTCGTATCCAGTGCAGGGTCCGAGGTATTCGCACTGGATACGACGCAAGC-3′ sense:5′-ACGGGGCTTTTTGCTGTCTGGG-3′ antisense:5′-CGCAGGGTCCGAGGTATTC-3′
hsa-let-7e-5p	MIMAT0000066	RT-premier:5′-GTCGTATCCAGTGCAGGGTCCGAGGTATTCGCACTGGATACGACAACTAT-3′ sense:5′-ACGGGCTGAGGTAGGAGGTTGT-3′ antisense:5′-CGCAGGGTCCGAGGTATTC-3′
hsa-miR-216a-5p	MIMAT0000273	RT-premier:5′-GTCGTATCCAGTGCAGGGTCCGAGGTATTCGCACTGGATACGACTCACAG-3′ sense:5′-ACGGGCTAATCTCAACTGGCAA-3′ antisense:5′-CGCAGGGTCCGAGGTATTC-3′
hsa-let-7c-5p	MIMAT0000064	RT-premier:5′-GTCGTATCCAGTGCAGGGTCCGAGGTATTCGCACTGGATACGACAACCAT-3′ sense:5′-ACGGGCTGAGGTAGTAGTTTGT-3′ antisense:5′-CGCAGGGTCCGAGGTATTC-3′
hsa-let-7b-5p	MIMAT0000063	RT-premier:5′-GTCGTATCCAGTGCAGGGTCCGAGGTATTCGCACTGGATACGACAACCAC-3′ sense:5′-ACGGGCTGAGGTAGTAGGGTGT-3′ antisense:5′-CGCAGGGTCCGAGGTATTC-3′
hsa-let-7g-5p	MIMAT0000414	RT-premier:5′-GTCGTATCCAGTGCAGGGTCCGAGGTATTCGCACTGGATACGACAACTGT-3′ sense:5′-ACGGGCTGAGGTAGTAGTTTGT-3′ antisense:5′-CGCAGGGTCCGAGGTATTC-3′
hsa-miR-335-5p	MIMAT0000765	RT-premier:5′-GTCGTATCCAGTGCAGGGTCCGAGGTATTCGCACTGGATACGACACATTT-3′ sense:5′-ACGGGGTCAAGAGCACTAACGA-3′ antisense:5′-CGCAGGGTCCGAGGTATTC-3′
hsa-let-7d-5p	MIMAT0000065	RT-premier:5′-GTCGTATCCAGTGCAGGGTCCGAGGTATTCGCACTGGATACGACAACTAT-3′ sense:5′-ACGGGGAGAGGTAGTAGGTGGC-3′ antisense:5′-CGCAGGGTCCGAGGTATTC-3′
hsa-miR-505-3p	MIMAT0002876	RT-premier:5′-GTCGTATCCAGTGCAGGGTCCGAGGTATTCGCACTGGATACGACAGGAAA-3′ sense:5′-ACGGGGCGTCAACACTGGCTGG-3′ antisense:5′-CGCAGGGTCCGAGGTATTC-3′
hsa-miR-107	MIMAT0000104	RT-premier:5′-GTCGTATCCAGTGCAGGGTCCGAGGTATTCGCACTGGATACGACTGATAG-3′ sense:5′-ACGGGCAGCAGCATTGTCCAGG-3′ antisense:5′-CGCAGGGTCCGAGGTATTC-3′
hsa-miR-495-3p	MIMAT0002817	RT-premier:5′-GTCGTATCCAGTGCAGGGTCCGAGGTATTCGCACTGGATACGACAAGAAG-3′ sense:5′-ACGGGTAAACAAACACGGGGCA-3′ antisense:5′-CGCAGGGTCCGAGGTATTC-3′
hsa-miR-324-5p	MIMAT0000761	RT-premier:5′-GTCGTATCCAGTGCAGGGTCCGAGGTATTCGCACTGGATACGACACACCA-3′ sense:5′-ACGGGTCGCATCACCGAGGGCA-3′ antisense:5′-CGCAGGGTCCGAGGTATTC-3′
hsa-miR-124-3p	MIMAT0000422	RT-premier:5′-GTCGTATCCAGTGCAGGGTCCGAGGTATTCGCACTGGATACGACGGCATT-3′ sense:5′-ACAGGCTAAGGCTCCCAGTGAA-3′ antisense:5′-CGCAGGGTCCGAGGTATTC-3′
hsa-miR-320b	MIMAT0005792	RT-premier:5′-GTCGTATCCAGTGCAGGGTCCGAGGTATTCGCACTGGATACGACTTGCCC-3′ sense:5′-ACGGGTAAAAGGTGGGTTGAGA-3′ antisense:5′-CGCAGGGTCCGAGGTATTC-3′
hsa-miR-146b-5p	MIMAT0002809	RT-premier:5′-GTCGTATCCAGTGCAGGGTCCGAGGTATTCGCACTGGATACGACAGCCTA-3′ sense:5′-ACGGTCTGACAACTGACTTCCA-3′ antisense:5′-CGCAGGGTCCGAGGTATTC-3′
hsa-miR-329-3p	MIMAT0001629	RT-premier:5′-GTCGTATCCAGTGCAGGGTCCGAGGTATTCGCACTGGATACGACAAAGAG-3′ sense:5′-ACGGGCATCACACTTGGCTAAC-3′ antisense:5′-CGCAGGGTCCGAGGTATTC-3′
hsa-miR-543	MIMAT0004954	RT-premier:5′-GTCGTATCCAGTGCAGGGTCCGAGGTATTCGCACTGGATACGACAAGAAG-3′ sense:5′-ACGGGTAAACATTCGTGGGGCA-3′ antisense:5′-CGCAGGGTCCGAGGTATTC-3′
hsa-miR-154-5p	MIMAT0000452	RT-premier:5′-GTCGTATCCAGTGCAGGGTCCGAGGTATTCGCACTGGATACGACCGAAGG-3′ sense:5′-ACCAGGTAGGTTATCCGTGTTG-3′ antisense:5′-CGCAGGGTCCGAGGTATTC-3′
hsa-miR-365a-3p	MIMAT0000710	RT-premier:5′-GTCGTATCCAGTGCAGGGTCCGAGGTATTCGCACTGGATACGACATAAGG-3′ sense:5′-ACGAGGTAATGCCGCTAAAAAT-3′ antisense:5′-CGCAGGGTCCGAGGTATTC-3′
hsa-miR-193a-3p	MIMAT0000459	RT-premier:5′-GTCGTATCCAGTGCAGGGTCCGAGGTATTCGCACTGGATACGACACTGGG-3′ sense:5′-ACGGGCAAGTGGGCTACAAAGT-3′ antisense:5′-CGCAGGGTCCGAGGTATTC-3′
hsa-miR-10a-5p	MIMAT0000253	RT-premier:5′-GTCGTATCCAGTGCAGGGTCCGAGGTATTCGCACTGGATACGACCACAAA-3′ sense:5′-AGCCGTTCCCGTGTAGAGCCGA-3′ antisense:5′-CGCAGGGTCCGAGGTATTC-3′
hsa-miR-27a-3p	MIMAT0000084	RT-premier:5′-GTCGTATCCAGTGCAGGGTCCGAGGTATTCGCACTGGATACGACGCGGAA-3′ sense:5′-ACGGGATTCACAGTGGCTAAGT-3′ antisense:5′-CGCAGGGTCCGAGGTATTC-3′
hsa-miR-181d-5p	MIMAT0002821	RT-premier:5′-GTCGTATCCAGTGCAGGGTCCGAGGTATTCGCACTGGATACGACACCCAC-3′ sense:5′-ACGGGTAAGATTCATTGTTGTC-3′ antisense:5′-CGCAGGGTCCGAGGTATTC-3′
hsa-miR-10b-5p	MIMAT0000254	RT-premier:5′-GTCGTATCCAGTGCAGGGTCCGAGGTATTCGCACTGGATACGACCACAAA-3′ sense:5′-AGGGATTGCCGTGTAGAACTGA-3′ antisense:5′-CGCAGGGTCCGAGGTATTC-3′
hsa-miR-499a-5p	MIMAT0002870	RT-premier:5′-GTCGTATCCAGTGCAGGGTCCGAGGTATTCGCACTGGATACGACAAACAT-3′ sense:5′-ACGGGATAAAGGCTTCCAGTGA-3′ antisense:5′-CGCAGGGTCCGAGGTATTC-3′
hsa-miR-320a	MIMAT0000510	RT-premier:5′-GTCGTATCCAGTGCAGGGTCCGAGGTATTCGCACTGGATACGACTCGCCC-3′ sense:5′-ACGGGTAAAAGGTGGGTTGAGA-3′ antisense:5′-CGCAGGGTCCGAGGTATTC-3′
hsa-miR-204-5p	MIMAT0000265	RT-premier:5′-GTCGTATCCAGTGCAGGGTCCGAGGTATTCGCACTGGATACGACAGGCAT-3′ sense:5′-ACGGGCTTCGCTTTGTCATTCT-3′ antisense:5′-CGCAGGGTCCGAGGTATTC-3′
hsa-miR-27b-3p	MIMAT0000419	RT-premier:5′-GTCGTATCCAGTGCAGGGTCCGAGGTATTCGCACTGGATACGACGCAGAA-3′ sense:5′-ACGGGGTTCACAGTGGCTAAGT-3′ antisense:5′-CGCAGGGTCCGAGGTATTC-3′
hsa-miR-504-5p	MIMAT0002875	RT-premier:5′-GTCGTATCCAGTGCAGGGTCCGAGGTATTCGCACTGGATACGACGATAGA-3′ sense:5′-AGAACGACACGCTGGTCTACAC-3′ antisense:5′-CGCAGGGTCCGAGGTATTC-3′
hsa-miR-9-5p	MIMAT0000441	RT-premier:5′-GTCGTATCCAGTGCAGGGTCCGAGGTATTCGCACTGGATACGACTCATAC-3′ sense:5′-ACGGTGTCTTTGGTTATCTGGC-3′ antisense:5′-CGCAGGGTCCGAGGTATTC-3′
hsa-miR-181b-5p	MIMAT0000257	RT-premier:5′-GTCGTATCCAGTGCAGGGTCCGAGGTATTCGCACTGGATACGACACCCAC-3′ sense:5′-ACGGCTAAGATTCATTGTTGTC-3′ antisense:5′-CGCAGGGTCCGAGGTATTC-3′
hsa-miR-194-5p	MIMAT0000460	RT-premier:5′-GTCGTATCCAGTGCAGGGTCCGAGGTATTCGCACTGGATACGACTCCACA-3′ sense:5′-ACAGCGTGTAACAGCATCTCCA-3′ antisense:5′-CGCAGGGTCCGAGGTATTC-3′
hsa-miR-708-5p	MIMAT0004926	RT-premier:5′-GTCGTATCCAGTGCAGGGTCCGAGGTATTCGCACTGGATACGACCCCAGC-3′ sense:5′-ACGGGCTAACGAGTTTACAATC-3′ antisense:5′-CGCAGGGTCCGAGGTATTC-3′
hsa-miR-34c-5p	MIMAT0000686	RT-premier:5′-GTCGTATCCAGTGCAGGGTCCGAGGTATTCGCACTGGATACGACGCAATC-3′ sense:5′-ACAGGCAGGCAGTGTAGTTAGC-3′ antisense:5′-CGCAGGGTCCGAGGTATTC-3′
hsa-miR-342-3p	MIMAT0000753	RT-premier:5′-GTCGTATCCAGTGCAGGGTCCGAGGTATTCGCACTGGATACGACACGGGT-3′ sense:5′-ACAGGCTCTCACACAGAAATCG-3′ antisense:5′-CGCAGGGTCCGAGGTATTC-3′
hsa-miR-377-3p	MIMAT0000730	RT-premier:5′-GTCGTATCCAGTGCAGGGTCCGAGGTATTCGCACTGGATACGACACAAAA-3′ sense:5′-ACGGGCATCACACAAAGGCAAC-3′ antisense:5′-CGCAGGGTCCGAGGTATTC-3′
hsa-miR-193b-3p	MIMAT0002819	RT-premier:5′-GTCGTATCCAGTGCAGGGTCCGAGGTATTCGCACTGGATACGACAGCGGG-3′ sense:5′-ACGGCTATCTGGTCCCCAAAGT-3′ antisense:5′-CGCAGGGTCCGAGGTATTC-3′
hsa-miR-181c-5p	MIMAT0000258	RT-premier:5′-GTCGTATCCAGTGCAGGGTCCGAGGTATTCGCACTGGATACGACACTCAC-3′ sense:5′-ACGGGCAAGATTCAACCAGACG-3′ antisense:5′-CGCAGGGTCCGAGGTATTC-3′
hsa-miR-125a-5p	MIMAT0000443	RT-premier:5′-GTCGTATCCAGTGCAGGGTCCGAGGTATTCGCACTGGATACGACTCACAG-3′ sense:5′-ACAGGCTCCTTGAGACGCTTTA-3′ antisense:5′-CGCAGGGTCCGAGGTATTC-3′
hsa-miR-103a-3p	MIMAT0000101	RT-premier:5′-GTCGTATCCAGTGCAGGGTCCGAGGTATTCGCACTGGATACGACTCATAG-3′ sense:5′-ACGGCTTAGCAGCATTGAACAG-3′ antisense:5′-CGCAGGGTCCGAGGTATTC-3′
hsa-miR-3619-5p	MIMAT0017999	RT-premier:5′-GTCGTATCCAGTGCAGGGTCCGAGGTATTCGCACTGGATACGACGCTGCA-3′ sense:5′-ACGGGCTCAACTGGAAGGCTGG-3′ antisense:5′-CGCAGGGTCCGAGGTATTC-3′
hsa-miR-146a-5p	MIMAT0000449	RT-premier:5′-GTCGTATCCAGTGCAGGGTCCGAGGTATTCGCACTGGATACGACAACCCA-3′ sense:5′-ACAGGCTGAGCACTGAGTTCCA-3′ antisense:5′-CGCAGGGTCCGAGGTATTC-3′
hsa-miR-339-5p	MIMAT0000764	RT-premier:5′-GTCGTATCCAGTGCAGGGTCCGAGGTATTCGCACTGGATACGACCGTGAG-3′ sense:5′-ACGGGCTACCAGTCATCAAGGA-3′ antisense:5′-CGCAGGGTCCGAGGTATTC-3′
hsa-miR-590-3p	MIMAT0004801	RT-premier:5′-GTCGTATCCAGTGCAGGGTCCGAGGTATTCGCACTGGATACGACACTAGC-3′ sense:5′-ACGGCGTAGTTTGATGTGTAAG-3′ antisense:5′-CGCAGGGTCCGAGGTATTC-3′
hsa-miR-383-5p	MIMAT0000738	RT-premier:5′-GTCGTATCCAGTGCAGGGTCCGAGGTATTCGCACTGGATACGACAGCCAC-3′ sense:5′-ACGGGCAGAGCAGGAGGTGATT-3′ antisense:5′-CGCAGGGTCCGAGGTATTC-3′
hsa-miR-506-3p	MIMAT0002878	RT-premier:5′-GTCGTATCCAGTGCAGGGTCCGAGGTATTCGCACTGGATACGACTCTACT-3′ sense:5′-ACTGGCTAAAGCACCATTCTGA-3′ antisense:5′-CGCAGGGTCCGAGGTATTC-3′
hsa-miR-34a-5p	MIMAT0000255	RT-premier:5′-GTCGTATCCAGTGCAGGGTCCGAGGTATTCGCACTGGATACGACACAACC-3′ sense:5′-ACAGCGTGGCAGTGTCTTATCT-3′ antisense:5′-CGCAGGGTCCGAGGTATTC-3′
hsa-miR-101-3p	MIMAT0000099	RT-premier:5′-GTCGTATCCAGTGCAGGGTCCGAGGTATTCGCACTGGATACGACACAACC-3′ sense:5′-ACTGGCTACGGTGCTGTGATAA-3′ antisense:5′-CGCAGGGTCCGAGGTATTC-3′
hsa-miR-181a-5p	MIMAT0000256	RT-premier:5′-GTCGTATCCAGTGCAGGGTCCGAGGTATTCGCACTGGATACGACACTCAC-3′ sense:5′-ACGGGCAAGATTCAACGCAGTC-3′ antisense:5′-CGCAGGGTCCGAGGTATTC-3′
hsa-miR-202-3p	MIMAT0002811	RT-premier:5′-GTCGTATCCAGTGCAGGGTCCGAGGTATTCGCACTGGATACGACTTCCCA-3′ sense:5′-ACAGGCAGAGGTATAGGGCAAG-3′ antisense:5′-CGCAGGGTCCGAGGTATTC-3′
hsa-miR-211-5p	MIMAT0000268	RT-premier:5′-GTCGTATCCAGTGCAGGGTCCGAGGTATTCGCACTGGATACGACAGGCGA-3′ sense:5′-ACGGGCTTCCATTTGTCATTCT-3′ antisense:5′-CGCAGGGTCCGAGGTATTC-3′
hsa-miR-22-3p	MIMAT0000077	RT-premier:5′-GTCGTATCCAGTGCAGGGTCCGAGGTATTCGCACTGGATACGACACAGTT-3′ sense:5′-ACGGCTAAGATGCCAGTTGAAG-3′ antisense:5′-CGCAGGGTCCGAGGTATTC-3′
hsa-miR-320c	MIMAT0005793	RT-premier:5′-GTCGTATCCAGTGCAGGGTCCGAGGTATTCGCACTGGATACGACACCCTC-3′ sense:5′-ACGGCTAAAAGCAGGGTTGAGA-3′ antisense:5′-CGCAGGGTCCGAGGTATTC-3′
hsa-miR-371a-5p	MIMAT0004687	RT-premier:5′-GTCGTATCCAGTGCAGGGTCCGAGGTATTCGCACTGGATACGACAGTGCC-3′ sense:5′-ACTGACACTCAAACTGTGGGGG-3′ antisense:5′-CGCAGGGTCCGAGGTATTC-3′
hsa-miR-3139	MIMAT0015007	RT-premier:5′-GTCGTATCCAGTGCAGGGTCCGAGGTATTCGCACTGGATACGACAACAGG-3′ sense:5′-ACGGCGTAGGAACTCAACAGAT-3′ antisense:5′-CGCAGGGTCCGAGGTATTC-3′
hsa-miR-449a	MIMAT0001541	RT-premier:5′-GTCGTATCCAGTGCAGGGTCCGAGGTATTCGCACTGGATACGACACCAGC-3′ sense:5′-ACGGGCTGGCAGTGTATTGTTA-3′ antisense:5′-CGCAGGGTCCGAGGTATTC-3′
hsa-miR-362-3p	MIMAT0004683	RT-premier:5′-GTCGTATCCAGTGCAGGGTCCGAGGTATTCGCACTGGATACGACTGAATC-3′ sense:5′-ACAGGCAACACACCGATTCAAG-3′ antisense:5′-CGCAGGGTCCGAGGTATTC-3′
hsa-miR-761	MIMAT0010364	RT-premier:5′-GTCGTATCCAGTGCAGGGTCCGAGGTATTCGCACTGGATACGACTGTGTC-3′ sense:5′-ACAGCGGCAGCAGGGTGAAACT-3′ antisense:5′-CGCAGGGTCCGAGGTATTC-3′
hsa-miR-425-5p	MIMAT0003393	RT-premier:5′-GTCGTATCCAGTGCAGGGTCCGAGGTATTCGCACTGGATACGACTCAACG-3′ sense:5′-ACAGGCAATCACACGAGCACTC-3′ antisense:5′-CGCAGGGTCCGAGGTATTC-3′
hsa-miR-449b-5p	MIMAT0003327	RT-premier:5′-GTCGTATCCAGTGCAGGGTCCGAGGTATTCGCACTGGATACGACGCCAGC-3′ sense:5′-ACGGGCAGGCAGTGTATTGTTA-3′ antisense:5′-CGCAGGGTCCGAGGTATTC-3′
hsa-miR-4500	MIMAT0019036	RT-premier:5′-GTCGTATCCAGTGCAGGGTCCGAGGTATTCGCACTGGATACGACAAGAAA-3′ sense:5′-ACGGGCTGAGGTAGTAGTTTCT-3′ antisense:5′-CGCAGGGTCCGAGGTATTC-3′
hsa-miR-3529-5p	MIMAT0019828	RT-premier:5′-GTCGTATCCAGTGCAGGGTCCGAGGTATTCGCACTGGATACGACAACAAC-3′ sense:5′-ACGGGCAGGTAGACTGAGATTT-3′ antisense:5′-CGCAGGGTCCGAGGTATTC-3′
hsa-miR-4725-5p	MIMAT0019843	RT-premier:5′-GTCGTATCCAGTGCAGGGTCCGAGGTATTCGCACTGGATACGACGGTGGG-3′ sense:5′-ACTATCAGCCCCTGTAGCCATC-3′ antisense:5′-CGCACGGTCCGAGGTATTC-3′
hsa-miR-4319	MIMAT0016870	RT-premier:5′-GTCGTATCCAGTGCAGGGTCCGAGGTATTCGCACTGGATACGACGTGGCT-3′ sense:5′-ACGACATCCCAGAGCAAAGCCA-3′ antisense:5′-CGCAGGGTCCGAGGTATTC-3′
hsa-miR-4429	MIMAT0018944	RT-premier:5′-GTCGTATCCAGTGCAGGGTCCGAGGTATTCGCACTGGATACGACCGCCTC-3′ sense:5′-ACGGGCAAAAGATGGGCTGAGA-3′ antisense:5′-CGCAGGGTCCGAGGTATTC-3′
hsa-miR-4262	MIMAT0016894	RT-premier:5′-GTCGTATCCAGTGCAGGGTCCGAGGTATTCGCACTGGATACGACCAGGTA-3′ sense:5′-ACGGGCGACATTCAGACTAACT-3′ antisense:5′-CGCAGGGTCCGAGGTATTC-3′
hsa-miR-4458	MIMAT0018980	RT-premier:5′-GTCGTATCCAGTGCAGGGTCCGAGGTATTCGCACTGGATACGACTTCTTC-3′ sense:5′-ACGGGCAGAGGTAGGTGTAGAA-3′ antisense:5′-CGCAGGGTCCGAGGTATTC-3′

### Pull-down assay with biotinylated miR-377-3p

A549 and H1299 cells were transiently transfected with biotinylated miR-377-3p, miR-377-3p-Mut and negative control of miR-377-3p (GenePharma, Shanghai, China), harvested and lysed 48 h after transfection. 50 μL of the samples were aliquoted for input. The remaining lysates were incubated with Dynabeads M-280 Streptavidin (Invitrogen, CA, USA) according to the manufacturer's protocol. In brief, the washed beads were treated in RNase-free solutions and incubated with equal volume of biotinylated miR-377-3p for 10 min at room temperature in binding and washing buffer on a rotator. Then, the beads with the immobilized miR-377-3p fragment were incubated with 10 mM EDTA pH 8.2 with 95% formamide at 65°C for 5 min. The bound RNAs were purified using Trizol for the qRT-PCR analysis.

### Pull-down assay with biotinylated DNA probe

The biotinylated DNA probe complementary to NEAT1 was synthesized (GenePharma, Shanghai, China), dissolved in binding and washing buffer, and incubated with Dynabeads M-280 Streptavidin (Invitrogen, CA, USA) at room temperature for 10 min to generate probe-coated beads according to the manufacturer's protocol. Then, A549 and H1299 cell lysates were incubated with the probe-coated beads, and the RNA complexes bound to these beads were eluted and extracted for qRT-PCR analysis. The NEAT1 pull-down probe sequence was 5′-Bio-GGTAAGTGAATTTTGTAATGGA-3′; and random pull down probe sequence used as negative control was 5′-Bio-ACCGTAACCAGACCCTTAGCCGAACC-3′.

### Colony formation assay

Cells were transfected with miR-377-3p mimic or miR mimic NC, miR-377-3p mimic+E2F3, SiRNA NEAT1 or scrambled, NEAT1 or NC, as described above. Twenty four hours later, transfected cells were trypsinized, counted and replated at a density of 500 cells/6 cm dish. Ten days later, colonies resulting from the surviving cells were fixed with 3.7% methanol, stained with 0.1% crystal violet and counted. Colonies containing at least 50 cells were scored. Each assay was performed in triplicates.

### Luciferase reporter assays

The 3′-UTR of human *E2F3 or lncRNA NEAT1* were amplified from human genomic DNA and individually inserted into the pmiR-RB-REPORT™ (Ribobio, Guangzhou, China) using the XhoI and NotI sites. Similarly, the fragment of *E2F3 or lncRNA NEAT1* 3′-UTR mutant was inserted into the pmiR-RB-REPORT™ control vector at the same sites. For reporter assays, A549 and H1299 cells were co-transfected with wild-type (mutant) reporter plasmid and miR-Ribo™ mimics (miR-Ribo™ negative control) using Lipofectamine 2000 (Invitrogen). Firefly and Renilla luciferase activities were measured in cell lysates using the Dual-Luciferase Reporter Assay system. Luciferase activity was measured forty eight hours post-transfection using dual-glo luciferase reporter system according to the manufacturer's instructions (Promega, Madison, WI, USA). Firefly luciferase units were normalized against Renilla luciferase units to control for transfection efficiency.

### Transwell migration/invasion assay

A549 and H1299 cells were grown in RPMI 1640 medium containing 10% fetal bovine serum to ~60% confluence and transfected with miR-377-3p mimic or miR mimic NC, miR-377-3p mimic+E2F3, SiRNA NEAT1 or scrambled, NEAT1 or NC. After twenty four hours, the cells were harvested by trypsinization and washed once with Hanks’ balanced salt solution (Invitrogen). To measure cell migration, 8-mm pore size culture inserts (Transwell; Costar, High Wycombe, UK) were placed into the wells of 24-well culture plates, separating the upper and the lower chambers. In the lower chamber, 500 μL of RPMI 1640 containing 10% FBS was added. Then, serum-free medium containing 5 × 10^4^ cells were added to the upper chamber for migration assays, whereas 1 × 10^5^ cells were used for matrigel invasion assays. After twenty four hours of incubation at 37°C with 5% CO_2_, the number of cells that had migrated through the pores was quantified by counting 10 independent visual fields under the microscope (Olympus) using a × 20 magnifications, and cell morphology was observed by staining with 0.1% crystal violet. Filters were washed thoroughly with 1 × PBS. Each experiment was performed at least three times.

### BrdU immunofluorescence assay

A549 and H1299 cells were seeded on sterile cover glasses placed in the 6-well plates. After transfection with miR-377-3p mimic or miR mimic NC, miR-377-3p mimic+E2F3, SiRNA NEAT1 or scrambled, NEAT1 or NC, for forty eight hours, the BrdU (5-bromo-2-deoxyuridine; Sigma) stock solution at 10 mg/mL in saline was diluted 1000 × in the culture medium and incubated for 60 min. After washing with PBS, cells were then fixed for 20 min in 4% paraformaldehyde (PFA) and permeabilized with 0.3% Triton X-100 for 10 min. After blocking with 10% goat serum in PBS for 1 h, cells were incubated with a primary rabbit antibody against BrdU (1:200, Abcam) over night at 4°C, and then incubated with the secondary antibody coupled to a fluorescent marker, Cy3, at room temperature for 2 h. After DAPI staining and PBS washing, the cover slips were mounted on to glass slides with anti-fade solution and visualized using a fluorescence microscope (Olympus 600 auto-biochemical analyzer, Tokyo, Japan) with Image-Pro Plus software for image analysis, and 10 microscopic fields were taken for calculating BrdU.

### CCK8 assay

Cell growth was measured using the cell proliferation reagent WST-8 (Roche Biochemicals, Mannheim, Germany). After plating cells in 96-well microtiter plates (Corning Costar, Corning, NY) at 1.0 × 10^3^ /well, 10 μL of CCK8 was added to each well at the time of harvest, according to the manufacturer's instructions. Two hours after adding CCK8, cellular viability was determined by measuring the absorbance of the converted dye at 450 nm.

### Flow cytometry

A549 and H1299 cells transfected with siRNA-NEAT1 and Scrambled, pGCMV/NEAT1 and NC, were trypsinized and resuspended in 1 × binding buffer at 1 × 10^6^ cells/mL. 100 μL of this cell suspension was incubated with 5 μL of FITC-Annexin V and 5 μL propridium iodide (PI) for 15 minutes in the dark. The reaction was terminated with the addition of 400 μL 1 × binding buffer and analyzed with (FACSCalibur using the CellQuest software (Becton Dickinson). FITC-Annexin V-positive and PI-negative cells were considered as apoptotic and the experiments were carried out in triplicates.

### Caspase-3/7 activity assay

The activity of caspase-3/7 was determined using the caspase-3/7 activity kit (Beyotime Institute of Biotechnology, Haimen, China). To evaluate the activity of caspase-3/7, cell lysates were prepared after their respective treatment with various designated treatments. Assays were performed on 96-well microtitre plates by incubating 10 μL protein of cell lysate per sample in 80 μL reaction buffer (1% NP-40, 20 mM Tris-HCl (pH 7.5), 137 mM Nad and 10% glycerol) containing 10 μL caspase-3/7 substrate (Ac-DEVD-pNA) (2 mM). Lysates were incubated at 37°C for 4 hours. Samples were measured with an ELISA reader at an absorbance of 405nm. The detail analysis procedure was described in the manufacturer's protocol.

### Tumor formation in BALB/c nude mice

BALB/c athymic nude mice (male, 4-6-weeks old and 16-20 g) were purchased from Hubei Research Center of Laboratory Animal (Wuhan, China). All animal experiments were carried out in accordance with the Guide for the Care and Use of Laboratory Animals of Wuhan University. To establish lung cancer xenograft model, 5 × 10^6^ shRNA-NEAT1-A549, pGCMV/NEAT1-A549 and NC-A549 cells were suspended in 100 μL phosphate-buffered saline and inoculated subcutaneously into the flanks of nude mice, the transplanted nude mice were divided into five groups: NC, NEAT1, sh-NEAT1, miR-377-3p, NEAT1+miR-377-3p (n=6 each). After 8 days of transplantation, miR-377-3p agomir (miR-377-3p) (GenePharma, Shanghai, China) was directly injected into the implanted tumors (miR-377-3p and NEAT1+miR-377-3p groups) at the dose of 1 nmol (in 20μL phosphate-buffered saline) per mouse every 4 days for eight times. The tumor size was monitored by measuring the length (L) and width (W) with calipers every 4 day, and the volumes were calculated using the formula: (L × W^2^)/2. Mice were killed by cervical dislocation after anaesthetized with 10% chloral hydrate in day 36, and the tumors were excised and snap-frozen for protein and RNA extraction.

### Statistical analysis

All experiments were repeated 3 times independently. The results are presented as the means ± standard error mean (SEM). A two-tailed paired t-test was performed using SPSS 19.0 software in order to detect significant differences in measured variables between groups. A value of P<0.05 was considered to indicate a statistically significant difference.

## Nonstandard abbreviations

lncRNA: long non-coding RNA
NSCLC: non-small cell lung cancer
SCLC: small cell lung cancer
ceRNA: competing endogenous RNA
miR-377-3p: hsa-miRNA-377-3p
IARC: International Agency for Research on Cancer
NEAT1: nuclear enriched abundant transcript 1
siRNAs: small interfering RNAs
3′-UTR: 3′-untranslated region
ERα: estrogen receptor alpha
BCL2: B-cell lymphoma-2
